# Immunohistochemical Evaluation of the Pathological Effects of Diabetes Mellitus on the Major Salivary Glands of Albino Rats

**DOI:** 10.1055/s-0042-1749159

**Published:** 2022-07-04

**Authors:** Mashael Saeed Alqahtani, Sherif Sayed Hassan

**Affiliations:** 1 Division of Oral Pathology, Department of Basic and Clinical Oral Sciences, Faculty of Dentistry, Umm Al-Qura University, Makkah, Kingdom of Saudi Arabia; 2 Department of Oral Biology and Dental Anatomy, Faculty of Dentistry, Al-Azhar University, Assiut, Egypt; 3 Division of Oral Biology, Department of Basic and Clinical Oral Sciences, Faculty of Dentistry, Umm Al-Qura University, Makkah, Kingdom of Saudi Arabia

**Keywords:** parotid gland, sublingual, submandibular, diabetes, cytokeratin 17

## Abstract

**Objectives** Diabetes mellitus is a notorious chronic disease characterized by hyperglycemia. Our study aimed to determine the expression of cytokeratin 17 (CK17) in all major salivary glands of diabetic albino rats to provide more information about the pathological effects of DM on the intracellular structures of the gland parenchyma.

**Materials and Methods** Twenty male adult albino rats were utilized in the experiment and divided into two equal groups, group 1 (control rats) and group 2 (diabetic rats). The animals were sacrificed 45 days after diabetes induction. The major salivary gland complex of all groups was dissected and prepared for evaluation by histological and immunohistochemical expression of CK17.

**Results** Histological results prove that the salivary gland parenchyma of diabetic group undergo gland atrophy characterized with the presence of degenerated acini, dilated duct system, and presence of duct-like structure with predominance of fibrous tissue compartment and discrete fat cells. Immunohistochemical expression of CK17 of major salivary gland of control group revealed negative to diffuse mild expression in all duct cells and some serous acinar cells, whereas mucous acini were negatively stained. On the other hand, major salivary gland parenchyma of diabetic group demonstrated mild to strong expression of duct cells more concentrated at their apical part with moderate to strong expression of some serous acini of diffuse type, whereas mucous acini of both submandibular gland and sublingual gland were negatively stained.

**Conclusion** The severity and prevalence of CK 17 in our results are predictive of the pathological influence of the DM that interferes with saliva production and/or secretion leading to dry mouth. The results also showed clear changes in the cytokeratin expression of diabetic sublingual salivary gland, although it had little effect in the routine histological study with hematoxylin and eosin, confirming that routine studies are not sufficient to form a definitive opinion.

## Introduction

### Salivary glands

Salivary glands are a group of both major and minor exocrine glands that drain saliva into the oral cavity. The salivary glands of rats consist of three pairs of major glands, parotid gland (PG), submandibular gland (SMG) and sublingual gland (SLG), as well as a lot of minor salivary glands. 1 Minor salivary glands are distributed throughout most parts of the oral cavity, and their secretions directly bathe the oral tissues. 2 Saliva that secreted by the acinar cells and modified by the duct cells, play an important role in maintaining the healthy state of the oral tissues, namely the teeth, gingiva and oral mucous membrane. 3 Saliva consists mainly of the secretions of SMG (65%), PG (23%), SLG (4%) and the remaining 8% being provided by the minor numerous glands. 1 Salivary secretion is composed mainly of water, electrolytes, and biologically active proteins, including growth factors and cytokines. 4 A series of salivary ducts was discovered in the seventeenth century by Nils Stensen (1638–1686), Thomas Wharton (1614–1673), and Caspar Bartholin (1655–1738) and through these ducts saliva pours into the oral cavity. 5 Saliva plays many diverse roles through its digestive, mastication, swallowing, antibacterial, buffering, lubricant, and water balance functions. 6

### Diabetes Mellitus

Diabetes mellitus (DM) is a generalized metabolic disease characterized by hyperglycemia results from abnormalities in carbohydrate metabolism. 7 The long-term prognosis of the diabetics is based on the consistency of residual fasting plasma glucose levels above 126 mg/dL. 8 According to the World Health Organization, the Kingdom of Saudi Arabia ranked second in terms of the incidence of DM in the Middle East countries, with seven million diabetics among its citizens. 9 10 Due to the high incidence of DM in humans, the induction of diabetes in animal models has been performed on a large scale to study its pathological effects on different organ systems. The most common alterations of DM at the oral and dental level, include periodontal disease, dental caries, looseness, tooth extraction, poor wound healing, 11 dry socket, 12 candidiasis, tongue disorders, 13 inability to eat, and taste disorders. 14 15 All of the above signs and symptoms are associated with dry mouth or hyposalivation, as diabetes is the most common metabolic disease that damages the salivary glands by altering their tissue structure and/or mechanism of salivary secretion. 16 17 Many authors state that the decreased salivary flow rate in diabetic patients is caused by increased frequent urination, this causes the extracellular fluid to drop notoriously, which the salivary glands need to produce saliva. 18 19

Morphologically, the parotid glands of diabetic animals were decreased in size and characterized by intracellular lipid accumulation in both acini and intralobular ducts. 20 Hand et al 1984 recorded the presence of small lipid droplets in the basal cytoplasm of acinar cells as the first detectable change for induced diabetes on the first day and peaked at 4.5 months, when acinar cells contained large lipid vacuoles. 21 Anderson et al 1994 reported that the diameter and number of granular ducts were reduced in diabetic animals, but acinar cells was only affected 6 months after the induction of diabetes. 22 Histochemical staining of the tissue suggested that the intracellular lipid within the acini was mainly a triglyceride which may accumulate by decreased utilization in the synthesis of secretory granules. 23 Also, Piras et al 2010 concluded that diabetes causes specific changes in secretory protein expression in human salivary glands, which contribute to the altered oral environment. 24

### Cytoskeleton

It is known that the cell cytoplasm contains a three-dimensional network of filaments forming the cytoskeleton which consists of three main types, microtubules, microfilaments, and intermediate filaments. Microtubules are approximately 25 nm in diameter, while microfilaments are approximately 4 to 6 nm in diameter. The intermediate filaments are approximately 6 to 10 nm in diameter, they are termed intermediate filaments because their diameter is between microtubules and microfibrils. 25 Herrmann and Aebi described that there are six major classes of intermediate filaments identified within the animal cells which are cytokeratin, vimentin, desmin, glial fibrillary acidic protein, neurofilaments, and nuclear lamin. 26 Cytokeratin intermediate filaments are a family of related proteins encoded by various genes and are found in many epithelial cells. The proposed functions of cytokeratin include: (1) maintaining the cellular structure, (2) connecting cells together, (3) facilitating the movement of cell organelles, (4) facilitating the transport of substances within the cell, (5) playing a key role in maintaining the tensile strength and integrity of the epithelial tissue, and (6) regulate cell proliferation and apoptosis. 27

### Immunohistochemistry

Immunohistochemistry is a technique for detecting an intracellular component (e.g., filaments) which act as antigen by injecting it to an animal which respond by the production antibodies to this specific antigen forming antigen-antibody reaction. After injection, the intracellular component (antigen) bears one or more antibody binding sites, which are highly specific regions called epitopes. The animal mounts humeral immune response to this specific antigen and produce antibody specific to this epitope termed polyclonal antibody which can be isolated from the animal. 28 Monoclonal antibodies are produced in the laboratory by cell culture methods. Cytokeratin constitute an important biomarker because they are stable, relatively resistant to hydrolysis, formalin-fixed and paraffin-embedded. Also, cytokeratin shows great fidelity in expression and is highly antigenic. 25 The distribution of cytokeratin 17 (CK17) in the normal PG parenchyma is associated with cells of the duct system, whereas serous acinar cells have little or no cytokeratin in their cytoplasm. 22

Our study aimed to determine the expression of CK17 within the parenchymal elements of major salivary glands of both normal and diabetic albino rats to provide more information about the effects of DM on salivary glands structure that led to xerostomia.

## Materials and Method

### Grouping

This research was conducted on twenty adult male albino rats (Sprague Dawley strain) with body weight ranging between 150–175 grams. All animals were housed in polycarbonate cages under 8 to 16 dark-light cycles. A mixture of hard and soft foods was given with unrestricted access to water. Rats were maintained in an animal health care facility under the supervision of the local ethical committee in a laboratory animal colony, Faculty of Veterinary Medicine, Cairo University, Cairo, Egypt. Rats were divided into two equal groups (control group one and diabetic group two).

### Induction of Diabetes Mellitus

Rats of group two (fasted 12 hours before) were intraperitoneally injected with a single dose of 150 mg/kg body weight of alloxan tetrahydrate (Sigma Chemical Company, St. Louis, Missouri, United States) dissolved in physiological solution saline (0.9% NaCl). Ten days later, blood glucose concentration was determined using enzymatic colorimetric test on the bases of trend reaction. Animals presented a glucose level at or above 200 mg/dl were included in the diabetic group. The diabetic rats maintained neither diet nor drug and feed like the control animals. Control rats were injected with sterile saline to mimic the prick injection with the diabetes group.

### Tissue Preparation

On the 45th day after diabetes induction, rats of both groups were sacrificed by anesthesia with diethyl ether. The salivary gland complex of each animal was cut into small portions (4 × 4 × 4 mm) and fixed in Bouin's fixative for 3 days. Fixed tissues were washed and then dried with ascending degrees of alcohol, cleaned in xylol, and infiltrated with molten paraffin wax to build up a block. Serial tissue sections with a thickness of 5 μm were mounted on a glass slide to be stained with hematoxylin and eosin for routine histological examination.

### Immunohistochemical Staining

The tissue sections for immunohistochemistry were mounted on special slide coated with polyL-lysine recommended for staining procedures that necessitate handling with a target retrieval solution. Paraffin sections (5 μm thick) were immersed in 0.3% HO/methanol for 30 minutes to block endogenous peroxidase action and rinsed with phosphate-buffered saline. Sections were incubated with anti-CK17 E3 monoclonal antibody on streptavidin biotin method and hematoxylin counter stain. The positive staining reaction appeared as brown staining which reflects the intracellular distribution of CK17 intermediate filaments within the tissue compartments. Tissue sections were evaluated semiquantitatively and grades as negative (0), weak (1), light (2), medium (3), and intense (4) staining.

### Statistical Analysis

Data analysis was done using the package of SPSS, version 23 (IBM Inc., Chicago, Illinois, United States). The quantitative data were calculated as mean, standard deviation, and ranges when their distribution found parametric by test of normality. The comparison between each two independent groups of the same gland (control vs. diabetic) was done by using an independent *t* -test for the equality of means and Levene's test for equality of variances. Therefore, the *p* -value was considered significant at a level ≤ 0.05.

## Results

### Histopathological Evaluation

The PG of control group revealed a parenchymal lobular tissue filled with closely packed serous acini and duct system. These parenchymal elements are supported by connective tissue stroma that divides the gland into lobes and lobules. The SMG of control group consist of predominant serous acini, as well as lesser number of mucous acini, normal branching duct system and granular convoluted tubules. The SLG of control group consist of predominant mucous acini, many covered with serous demilune and few serous spherical acini with normal branching ducts system.

**Fig. 1:**
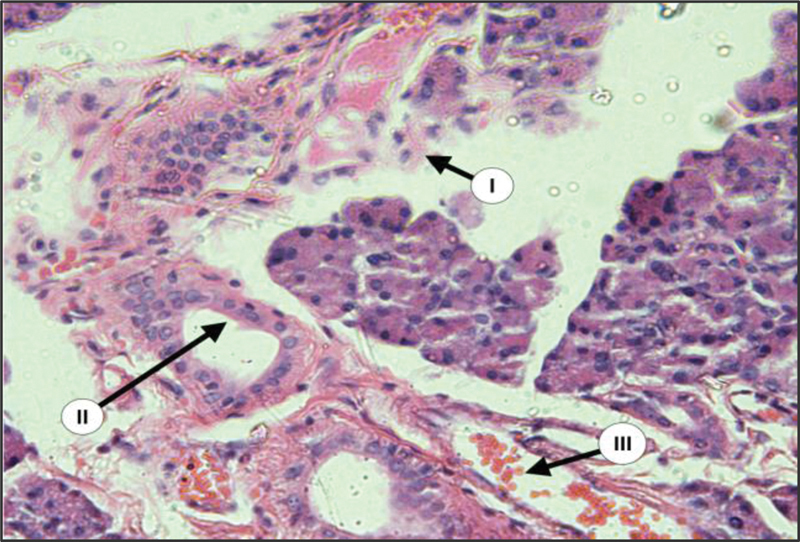
Parotid gland (PG) of diabetic rats showed loss of gland architecture with degenerated acini (I), dilated ducts (II), and dilated blood vessels (III) {H&E × 200}.

**Fig. 2:**
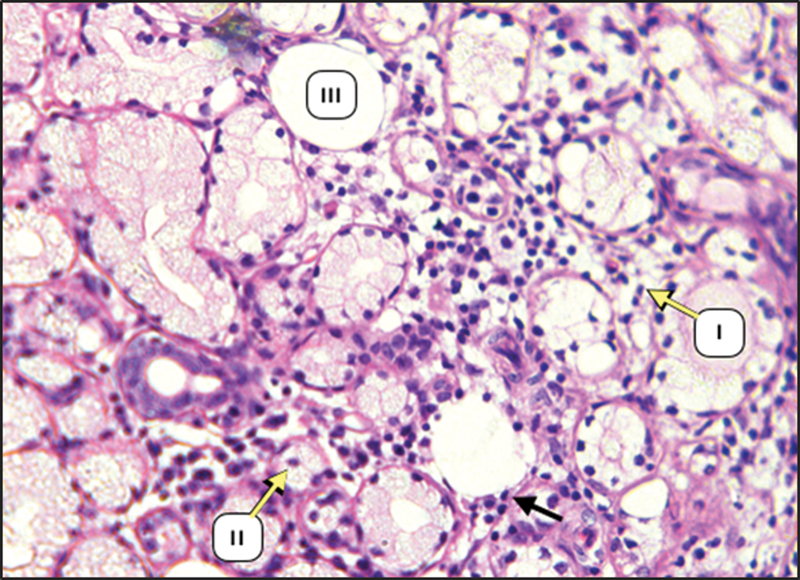
Submandibular gland (SMG) of diabetic rats showing acinar atrophy (I), decrease in the acinar size (II), fatty tissue (III) {H&E × 200}.

**Fig. 3:**
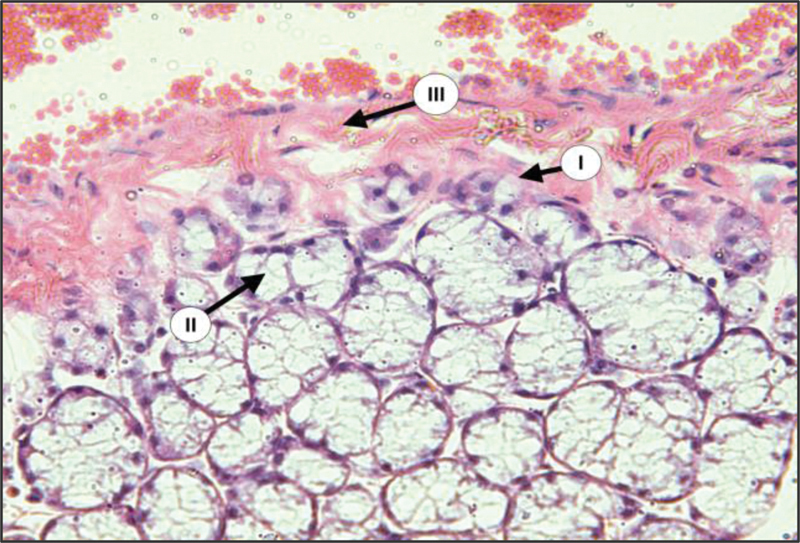
Sublingual gland (SLG) of diabetic rats showing serous acinar atrophy (I), decrease in the gland size (II), fibrous tissue (III) {H&E × 200}.

During surgery, there is a great reduction in the size of salivary gland complex of diabetic group in relation to the control group. The glandular elements of diabetic group revealed atrophic changes characterized by decrease in the parenchymal elements of all major glands accompanied by increase in the amount of fibrous stroma in both PG and SMG ( Figs. 1 , 2 , 3 ). The parenchymal elements consist of small serous acini with unspecified lumen. Both SMG and SLG showed an increase in the mucous acini among the persisted serous one. The duct systems showed an enlarged and dilated lumens with the presence of a duct-like structure. Moreover, many acini have been replaced by adipose tissue ( Fig. 1 ).

### Immunohistochemical Evaluation

**Fig. 4:**
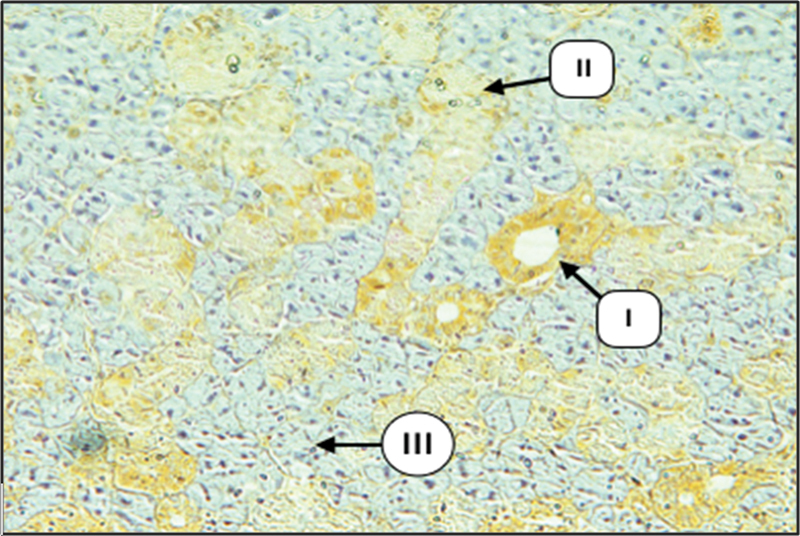
Submandibular gland (SMG) of control group showing mild expression of CK17 in duct cells (I), weak in GCT (II), and negative in serous acini (III) (× 100).

**Fig. 5:**
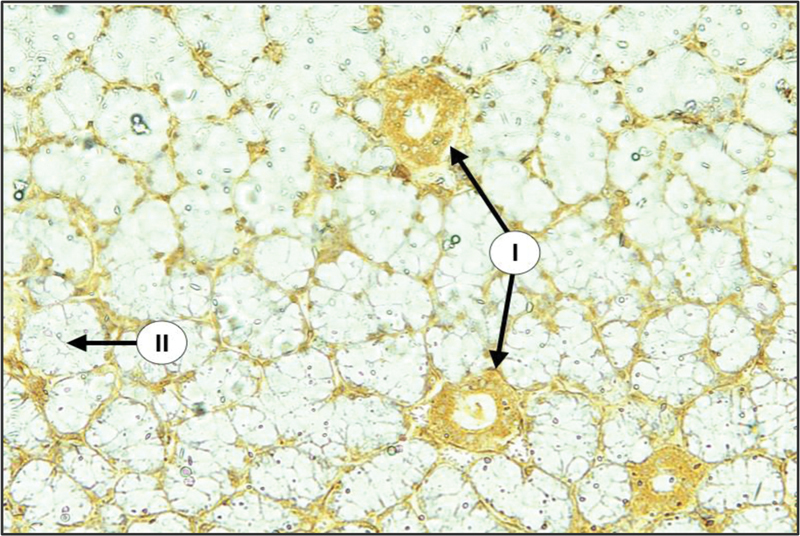
Sublingual gland (SLG) of control group showing mild expression of CK17 in duct cels (I), negative in mucous acini (II) (× 100).

Examination of major salivary glands of the control group incubated with anti-cytokeratin E3 antibody against CK17 using immunoperoxidase technique revealed that the duct cells showed diffuse weak to mild expression of CK17 ( Figs. 4 and 5 ). In some sections, both intercalated and striated ducts showed weak expression at the luminal part of the cells with moderate expression at their basal part. Some serous acini and serous demilune of mixed acini showed diffuse weak to mild expression of CK17, whereas the mucous acinar cells showed negative expression.

**Fig. 6:**
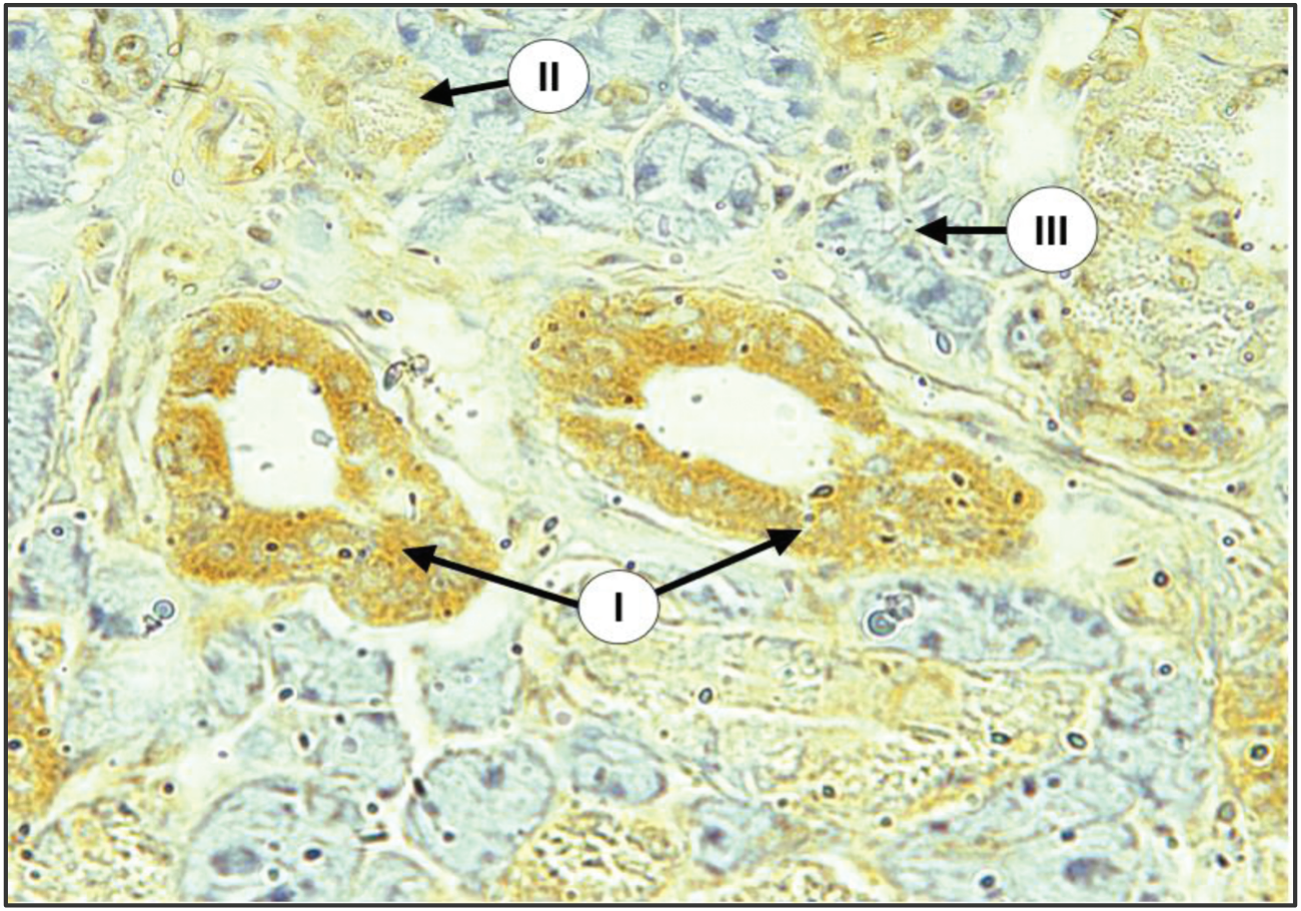
Submandibular gland (SMG) of diabetic group showing severe expression of CK17 in duct system (I), weak in some serous acini (II), and negative in most serous acini (III) (× 200).

**Fig. 7:**
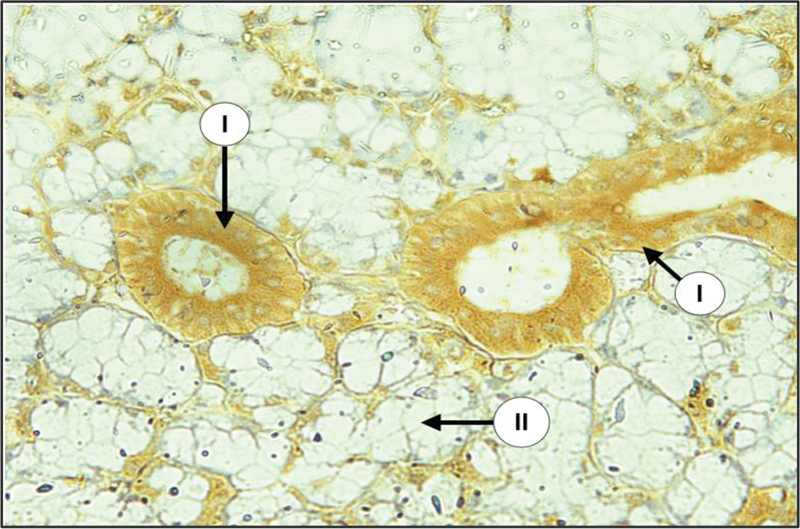
Sublingual gland (SLG) of diabetic group with severe expression of CK17 in duct cells (I), and negative in mucous acini (II) (× 200).

Examination of major salivary glands of the diabetic group revealed that both intercalated and striated duct cells displayed mild to strong cytoplasmic expression of CK17, the staining pattern was either strong at the apical part of cytoplasm with mild staining at their basal part or diffused throughout the cell cytoplasm. The main excretory ducts lined by stratified squamous epithelium demonstrated strong expression at the luminal cell layer with mild to moderate expression at the remaining layers. Granular convoluted tubular cells in SMG showed mild to moderate staining reaction of diffused pattern. Many serous acini revealed a mild to moderate expression of CK17 of diffuse type, whereas mucous acini were negatively stained in both SMG and SLG ( Figs. 6 and 7 ).

**Fig. 8:**
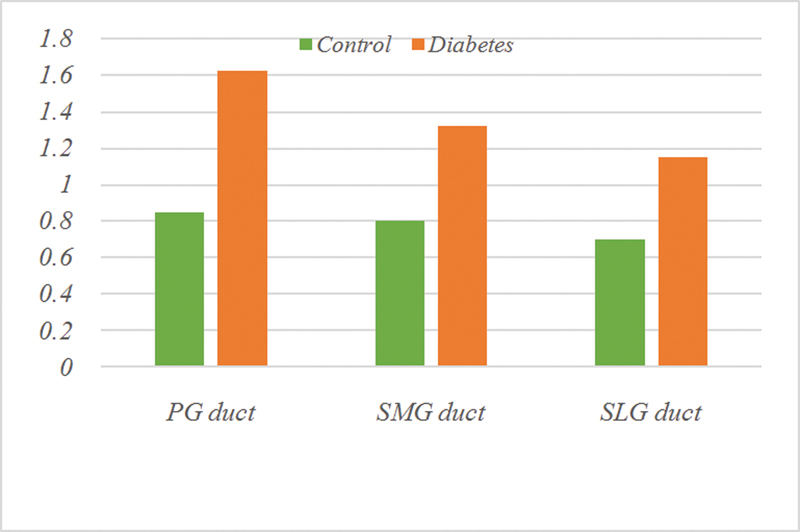
Expression of cytokeratin 17 (CK17) in duct cells of all groups.

The results of the statistical studies indicated that there were statistically significant differences between the concerned groups ( Tables 1 2 3 4 ). The most extreme of these differences were in duct cells PG (= 0.004) followed by duct cells from diabetic SMG (= 0.008), while the least effects were on acinar cells from diabetic SMG (= 0.036) ( Fig. 8 9 ).

**Table 1:** Group statistics of both control and diabetic rats

**Group statistics**											
	**Group**	***N***	**Mean**	**Standard deviation**	**Standard error mean**		**Group**	***N***	**Mean**	**Standard deviation**	**Standard error mean**
CK17 in PG duct	Control	10	0.8750	0.46022	0.14554	CK17 In PG acini	Control	10	0.6000	0.37639	0.11902
	Diabetic	10	1.5500	0.45338	0.14337		Diabetic	10	0.9750	0.29930	0.09465
CK17 in SMG duct	Control	10	0.8010	0.42218	0.13350	CK17 in SMG acini	Control	10	0.5250	0.36232	0.11457
	Diabetic	10	1.3250	0.35453	0.11211		Diabetic	10	0.8500	0.26882	0.08501
CK17 in SLG duct	Control	10	0.7010	0.35057	0.11086	CK17 in SLG acini	Control	10	0.6000	0.35749	0.11305
	Diabetic	10	1.1500	0.33751	0.10673		Diabetic	10	0.9740	0.32163	0.10171

Abbreviations: CK17, cytokeratin 17; PG, parotid gland; SLG, sublingual gland; SMG, submandibular gland.

**Table 2:** Independent samples test of CK 17 in parotid glands

		Levene's Test for Equality of Variances		t-test for Equality of Means						
		F	Sig.	t	Df	Sig. (2-tailed)	Mean Diff	Std. Error Diff	95% Confidence Interval of the Difference	
									Lower	Upper
CK17 in parotid duct	Equal variances assumed	0.274	0.607	3.304	18	0.004*	0.6750	0.20429	0.24579	1.10421
	Equal variances not assumed			3.304	17.996	0.004*	0.6750	0.20429	0.24579	1.10421
CK17 in parotid acini	Equal variances assumed	0.004	0.949	−2.466	18	0.024*	−0.3750	0.15207	−0.69449	−0.05551
	Equal variances not assumed			−2.466	17.131	0.025*	−0.3750	0.15207	−0.69565	−0.05435

Abbreviations: CK, cytokeratin; df, degrees of freedom. *Significant at a level 0.05.

**Table 3:** Independent samples test of CK 17 in submandibular glands

		Levene's Test for Equality of Variances		t-test for Equality of Means						
		F	Sig.	t	Df	Sig. (2-tailed)	Mean Diff	Std. Error Diff	95% Confidence Interval of the Difference	
									Lower	Upper
CK17 in SMG duct	Equal variances assumed	0.117	0.737	−3.006	18	0.008*	−0.5240	0.17433	−0.89026	−0.15774
	Equal variances not assumed			−3.006	17.478	0.008*	−0.5240	0.17433	−0.89105	−0.15695
CK17 in SMG acini	Equal variances assumed	0.889	0.358	−2.278	18	0.035*	−0.3250	0.14267	−0.62473	−0.02527
	Equal variances not assumed			−2.278	16.605	0.036*	−0.3250	0.14267	−0.62655	−0.02345

Abbreviations: CK, cytokeratin; df, degrees of freedom; SMG, submandibular gland. *Significant at a level 0.05.

**Table 4:** Independent samples test of CK17 in sublingual glands

		Levene's Test for Equality of Variances		t-test for Equality of Means						
		F	Sig.	t	Df	Sig. (2-tailed)	Mean Diff	Std. Error Diff	95% Confidence Interval of the Difference	
									Lower	Upper
CK17 in SLG duct	Equal variances assumed	0.622	0.440	−2.918	18	0.009*	−0.4490	0.15389	−0.77230	−0.12570
	Equal variances not assumed			−2.918	17.974	0.009*	−0.4490	0.15389	−0.77234	−0.12566
CK17 in SLG acini	Equal variances assumed	0.250	0.623	−2.459	18	0.024*	−0.3740	0.15207	−0.69348	−0.05452
	Equal variances not assumed			−2.459	17.803	0.024*	−0.3740	0.15207	−0.69374	−0.05426

Abbreviations: CK, cytokeratin; df, degrees of freedom; SLG, sublingual gland. *Significant at a level 0.05.

**Fig. 9:**
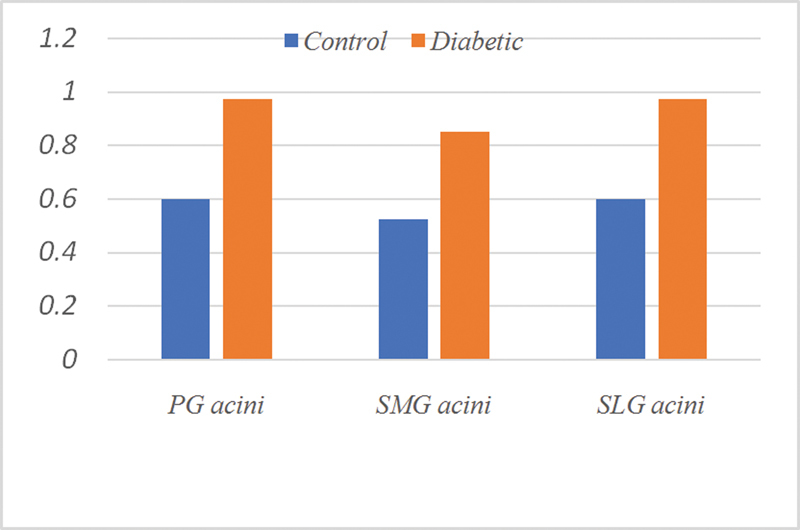
Expression of cytokeratin 17 (CK17) in acinar cells of all groups.

## Discussion

In general, damage to the major salivary glands is a known consequence of DM in both human and experimental models. Caldeira et al 2005) noted that Morphological changes in salivary gland are detected not only in uncontrolled diabetes but also in glycemic control. 17 Our results were recorded once on the forty-fifth day after confirming the occurrence of DM, and the results were evident in both acinar and ductal cells, in contrast to what Anderson and others said in 1994 that the gland acini was not affected until six months after induction of diabetes. 22 The results of the current study reported that DM caused structural changes ranging from a reduction in the acinar volume to severe atrophy of the gland parenchyma which was replaced by either fibrous or fatty tissue with proliferation of duct-like structures, this findings explain the occurrence of dry mouth with failure to perform the secretory activity. In the opposite direction to the atrophic changes of the parenchymal elements, the fibrous stroma interacts through a proliferative activity, illustrating the differences in tissue interaction of both epithelial and connective tissues. Anderson and Suleiman, (1989) suggest that the replacement of parenchymal cells with fibrous connective tissue is difficult to reconcile with the normal physiological responsiveness of the gland. 23 The fibrous tissue that replaced the degraded gland components in both PG and SMG appeared very extensive suggesting permanent changes with the glands unable to regenerate later. Contrary to our interpretation, Mata et al reported that persistent acini found in glandular tissues have been suggested to be involved in the gland's ability to regenerate. 16 In several samples of diabetic group, the presence of several normal and diminished acini indicates that the gland is still performing its secretory capacity but to a minimal degree. The results of our study are unable to make any attempt to distinguish between duct-like structures and duct system. This result was supported by Takahashi et al who reported that duct-like structures appear to be increased due to the proliferative activity of duct system cells. 31

All major salivary glands of the control group revealed CK17 expression with moderate intensity within the duct cells, while the serous acini showed weak expression and negative in the mucous acini as reported by Makino et al. 32 These observations may be due to highly differentiated acinar cells with a reduced amount of filamentous structure. Several authors agree with this finding that CK17 in salivary gland cells plays an important role in cell structure and the intensity of expression is closely related to the differentiation status of the parenchymal cells. 10 28 The different patterns of CK17 distribution are thought to be related to the functional activity of the gland where the diffuse pattern of staining indicates the nonsecretory state, while the decrease of CK17 in the luminal portion was associated with the active state of secretion leaving the area for exocytosis. The expression pattern of CK17 focused at the basal cell part may be associated with an increase in the tensile force of acinar cells facing myoepithelium resulting in an increased pressure capacity to drive saliva through the lumen into the duct system. The salivary glands of the diabetic group revealed significant CK17 staining in both acinar and ductal cells with two different appearances, lumen center or diffuse, which is opposite to the normal, dominant distribution of the control group. It is thought that both patterns of CK17 distribution may interfere with the secretory capacity of acinar cells resulting in xerostomia. Also, the luminal pattern within the duct cells may disturb the modulation procedure for primary secreted saliva. On the other hand, the diffuse pattern of CK17 indicates a cellular deleterious effect throughout acinar or ductal cells leading to apoptosis.
